# Theoretical Estimation of Thermal Effects in Drilling of Woven Carbon Fiber Composite

**DOI:** 10.3390/ma7064442

**Published:** 2014-06-12

**Authors:** José Díaz-Álvarez, Alvaro Olmedo, Carlos Santiuste, María Henar Miguélez

**Affiliations:** 1 Department of Bioengineering and Aerospace Engineering, Universidad Carlos III de Madrid, Avda. Universidad 30, Leganés, Madrid 28911, Spain; E-Mail: jodiaz@ing.uc3m.es; 2 Department of Continuum Media and Structural Analysis, Universidad Carlos III de Madrid, Avda. Universidad 30, Leganés, Madrid 28911, Spain; E-Mails: aolmedo@ing.uc3m.es (A.O.); csantius@ing.uc3m.es (C.S.); 3 Department of Mechanical Engineering, Universidad Carlos III de Madrid, Avda. Universidad 30, Leganés, Madrid 28911, Spain

**Keywords:** Carbon Fiber Reinforced Polymer (CFRPs), drilling, modeling, thermal effects

## Abstract

Carbon Fiber Reinforced Polymer (CFRPs) composites are extensively used in structural applications due to their attractive properties. Although the components are usually made near net shape, machining processes are needed to achieve dimensional tolerance and assembly requirements. Drilling is a common operation required for further mechanical joining of the components. CFRPs are vulnerable to processing induced damage; mainly delamination, fiber pull-out, and thermal degradation, drilling induced defects being one of the main causes of component rejection during manufacturing processes. Despite the importance of analyzing thermal phenomena involved in the machining of composites, only few authors have focused their attention on this problem, most of them using an experimental approach. The temperature at the workpiece could affect surface quality of the component and its measurement during processing is difficult. The estimation of the amount of heat generated during drilling is important; however, numerical modeling of drilling processes involves a high computational cost. This paper presents a combined approach to thermal analysis of composite drilling, using both an analytical estimation of heat generated during drilling and numerical modeling for heat propagation. Promising results for indirect detection of risk of thermal damage, through the measurement of thrust force and cutting torque, are obtained.

## 1. Introduction

The attractive properties of Carbon Fiber Reinforced Polymer (CFRPs) make this family of materials suitable for a wide range of high responsibility structural applications. CFRPs exhibit fatigue and corrosion resistance combined with lightweight, high specific stiffness, and strength. [1] CFRP components are manufactured to be near net shape, however, dimensional and assembly requirements commonly involve some machining operations. Mainly trimming and drilling are required, being critical operations, since they are performed on high value components susceptible to suffering machining induced damage. Delamination, fiber pull-out, and thermal degradation [2], usually observed when machining with worn tools or inappropriate cutting parameters, could affect the performance of the composite component or the mechanical joint during service life [3,4,5].

Delamination related to further strength reduction of the component, has received considerable attention in the scientific literature, using experimental and numerical approaches, see for instance recent advances in [6,7]. However, mechanical delamination is not the unique risk for the surface integrity of the component. Thermal damage, related to low glass transition temperatures (around 180 °C for a typical epoxy resin in CFRPs), can cause matrix degradation and, thus, it is also involved in plies separation [8].

Despite of the potential risk of thermal damage when machining CFRPs, it has been analyzed, mostly based on experimental works, and only in a few works in the literature. The measurement of the temperature at the cutting tool has been achieved, using thermocoupling, by several authors. Chen [9] obtained the temperature reached during drilling at the flank surface. A significant influence of the cutting speed was observed, with temperature increasing from 120 to 300 °C when the cutting speed increased from 40 to 200 m/min. Brinksmeier *et al.* [10] embedded a thermocouple at the tool tip for temperature measurement in drilling and orbital milling of hybrid components Ti/CFRP/Al. Drilling operation involved lower surface quality and higher temperatures than orbital milling.

The installation of a thermocouple inside the tool gives indirect information about the temperature level at the workpiece. Direct measurement of workpiece temperature was achieved in [8], also completed with temperature measurement at the cutting tool. Milling of CFRP was conducted with carbide endmill in dry conditions. Two different techniques were used: infrared thermo-graph camera for endmill surface temperature measurement, and embedded K-type thermocouple in the CFRP for measurement of the temperature at the machined surface. The temperature at a depth of 0.3 mm beneath the machined surface reached 104 °C (at cutting a speed equal to 300 m/min), this temperature being much lower than that measured at tool–chip contact point.

The influence of tool wear in temperature induced during trimming was analyzed in [11]. Fresh tools induced temperatures below the glass transition temperature of the composite, while a critical level was reached using worn tools. In addition, machining parameters had significant influence on the variation of the machined surface quality and cutting forces.

Cutting temperature in rotary ultrasonic machining of carbon fiber reinforced plastic has been recently analyzed [12] using two techniques: fiber optic sensor and thermocouples. Relations between input variables (ultrasonic power, tool rotation speed, and feed rate) and cutting temperature were obtained from experiments. The authors found that the maximum cutting temperature decreased as ultrasonic power and feed rate decreased. On the other hand, as tool rotation speed increased, maximum cutting temperature firstly increased, and then decreased. Concerning the method for temperature measurement, the fiber optic sensor gave higher temperatures than those measured by the thermocouple method.

The development of modeling tools, able to predict temperature distribution during composite cutting, is a desirable objective because of its relation with damage. Prediction of mechanical damage in composite cutting has been commonly achieved using finite element analyses. Although this field is not as active as metal cutting, it is possible to find some works focusing on orthogonal cutting of composite, involving two-dimensional (2D) models (see for instance [13,14,15,16]), as well as three-dimensional (3D) approaches (see, for example, [17,18] analyzing the validity of 2D hypotheses). Main contributions in the field of modeling of composite machining have been summarized in a recent review [19].

Simulation of real cutting processes, such as drilling, requires a high computational cost because of the need of simulating tool rotation and feed movement using both damage and failure criteria of the workpiece. These types of complex models for drilling have been recently developed showing good correlation between measured and predicted torque, thrust force, and delamination area [7,20].

Although mechanical effects in composite cutting have been analyzed using simulation tools, thermal effects have been neglected in these models. The first approach to the numerical modeling of thermal phenomena involved in the orthogonal cutting of CFRPs has been presented in a recent work of the authors [21]. The model included heat generation due to friction at the chip/tool interface and it was used for the prediction of intralaminar damage, delamination, and thermal damage accounted in terms of temperature level beneath the machined surface.

The heat generated due to plastic work can be assumed to be negligible in CFRPs because they exhibit elevated elastic modulus with small deformations, even when breakage is initiated. Deformation energy is negligible and, thus, heat generated can be neglected. This behavior is quite different from that observed in metal cutting, involving large amount of plastic work converted into heat, leading to very high temperatures at the primary shear zone and, sometimes, to the formation of adiabatic shear bands (see, for instance, [22]). As deformation energy is neglected, the unique heat source considered in the model was friction at the interface. Thus, the estimation of friction heat at the tool/chip interface is crucial in drilling. Measurement of temperature during industrial drilling is not possible since the thermocouple technique is invasive.

**Figure 1:**
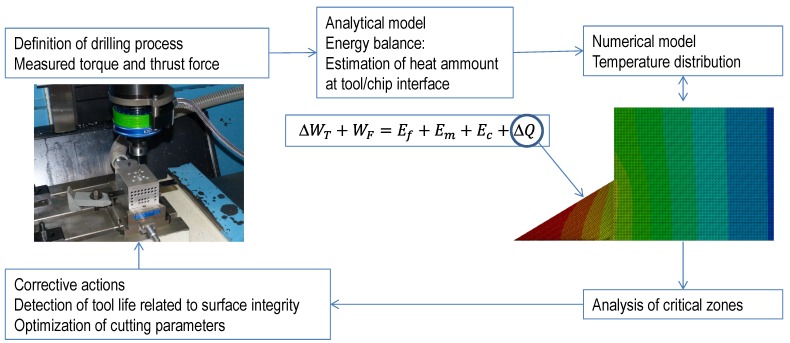
Relationships between experimental, analytical, and numerical steps proposed.

The main objective of this paper is the estimation of heat amount from easy-to-measure, in-process variables: torque and thrust force. A simple analysis, based on energy balance, is presented to obtain the heat generated at the interface, indirectly, from experimental tests. As far as the bibliographic revision has been carried out, this approach has not been applied to the problem of drilling. However, it is possible to find interesting analytical models of composite impact in [23], based on energy balance (impact processes have common characteristics with drilling, mainly the penetration of the projectile/drill into the target/workpiece). Once the frictional heat amount is available, it is possible to establish temperature distribution with a simple numerical model accounting for thermal conductivity in a composite. The detection of critical levels of temperature at certain zones can be used for assessment during the definition of drilling process of structural components, avoiding risk of thermal damage. The establishment of maximum levels of thermal energy, directly related with torque and thrust force evolution registered during cutting, could be used as an indicator of excessive tool wear. The aim of the paper and its practical application are illustrated in Figure 1.

This work is structured firstly in this introduction, followed by the estimation of heat developed in the second section. The third section involves the simulation of heat propagation, analyzing different cases and, finally, discussion and conclusions are presented.

## 2. Estimation of Frictional Heat

**Figure 2:**
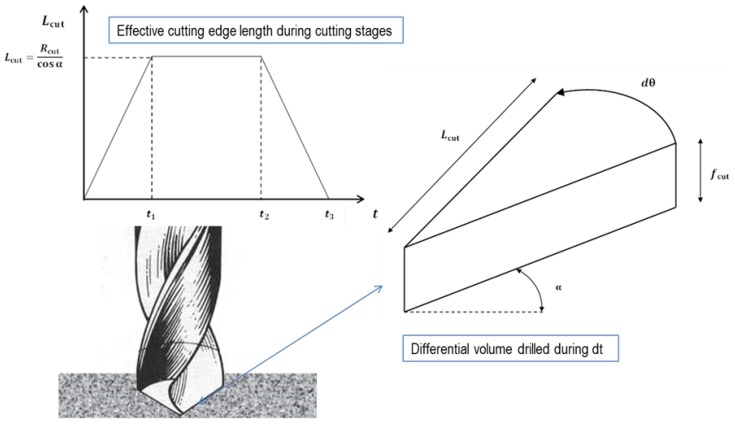
Scheme of drilling process: differential volume removed during the differential time d*t* and effective cutting edge at the different stages of drilling from entrance to drill exit.

A drilling process is performed at a constant feed and rotary velocity. The control of the machine tool maintains these parameters and the resultant torque and thrust force measured at the spindle are dependent on the cutting parameters, the material properties, and the characteristics of the cutting tool, including contact behavior at the interface. Figure 2 illustrates the entrance of the drill through the composite during cutting. Thus, a simplified method to estimate the heat generated at the interface has been developed assuming constant feed and rotary velocity of the drill. The assumption of axial-symmetry was considered. Although CFRP strength is orientation dependent, a woven laminate was selected to minimize the effect of the anisotropy.

### 2.1. Energy Balance

The differential work due to torque (d*W_T_*) and thrust force (d*W_F_*) during a differential time increment, d*t*, involved in a differential of turn angle of the drill, dθ, and is the result of several contributions. These contributions are summarized in Equation (1): the energy required for breakage of composite (d*E_f_*), the kinetic energy transferred to the chip (d*E_c_*), and the amount of heat generated at the interface due to friction (d*Q*).

d*W_T_* + d*W_F_* = d*E_f_* + d*E_c_ +* d*Q* (1)

The kinetic energy of the chip, once separated from the workpiece, can be neglected due to the small mass of the chip and the moderated velocity involved in cutting. A first estimation showed that the level was negligible compared to the rest of terms of Equation (1) (around 0.005% of the energy required for breakage of composite (d*E_f_*)). Thus, the heat generated at the interface can be estimated as:

d*Q =* d*W_T_* + d*W_F_* − d*E_f_* (2)

The terms corresponding to work developed by torque and thrust force were obtained from experiments. The torque and thrust force recorded at each time increment were derived from the discretization of the evolution with cutting time of both variables, measured during the drilling test.

The term corresponding to energy involved in composite breakage can be calculated considering the differential volume removed by the drill during a differential time, *dt*, corresponding to a differential drill turn, dθ.

d*E_f_*(θ) = *w_f_*d*V_f_*(θ) (3)

where *w_f_* is the specific energy of the woven composite breakage; and d*V_f_* is the differential volume associated to a differential turn of the drill, *d*θ.

The specific energy can be estimated as *w_f_* = 2(1/2*X*ε*_f_*), where *X* is the strength of the woven composite (the same in direction 1 and 2 because of the woven architecture of the composite); and ε*_f_* is the ultimate strain of the composite. It is worth noting that composite strength is orientation dependent, but the hypothesis of axial-symmetry was necessary to avoid unaffordable computational costs. The approach was similar to that used by Artero-Guerrero *et al.* [24] when modeling impact on a woven composite. Mechanical and thermal problems are uncoupled in the present, simplified model. Thus, mechanical properties are considered temperature independent under 180 °C, and thermal damage is assumed for temperatures above 180 °C.

The differential volume considered is presented in Figure 2. From this figure the volume can be calculated as:

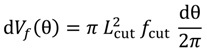
(4)
where *L*_cut_ is the effective cutting edge length; and *f*_cut_ is the feed rate.

It is worth noting that the volume of material removed depends on the stage of the drilling process. It is possible to distinguish three different stages illustrated in Figure 2, the entrance of the conical zone, the cut performed with the complete edge and the exit of the drill.

For the geometry of the drill used in the machining tests (described in the next section) the stages indicated in the figure corresponds with the following values of time and effective length of cutting edge.

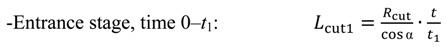
(5)

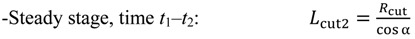
(6)

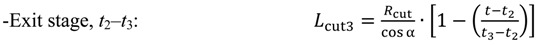
(7)
where *R*_cut_ is the drill radius.

The expression presented in Equation (2) was applied to real experiments involving drilling of woven carbon composite. The specific conditions of the experiments are presented in the next subsection, involving both new and worn tool geometries.

### 2.2. Application to Experiments

In order to apply the energy balance formulated in Equation (2) to a real case of drilling, experimental tests were performed on woven CFRP composite. Each ply was manufactured by Hexcel Composites consisting of AS-4 carbon fiber and epoxy matrix. The specimens, with a stacking sequence of 10 plies with the same fiber orientation in all of them, and a total thickness of 2.2 mm, were cut in plates. The mechanical properties of this material were obtained from scientific literature [24], see Table 1.

**Figure 3:**
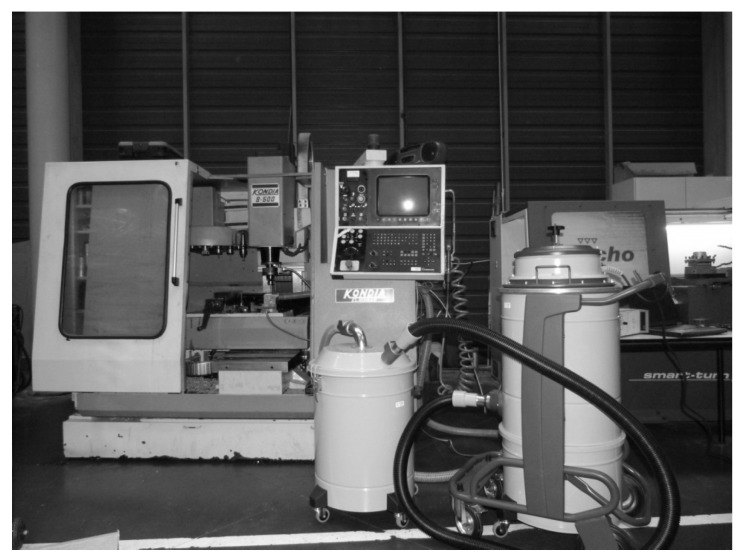
Machining center used in the experiments was equipped with the dynamometer, acquisition system and also with a system for chip aspiration.

The cutting tests were carried out in a machining center (B500 KONDIA, Kondia, Elgoibar, Spain), shown in Figure 3. The machining center was equipped with a dynamometer (Kistler 9123C, Winterthur, Switzerland) for measurement of cutting forces and torque (see Figure 3).

The drill (diameter, 6 mm; point angle, 118°) was recommended by the manufacturer GUHRING (Albstadt-Ebingen, Germany) for CFRP drilling.

Drilling tests were performed with new drill and with worn tool exhibiting flank wear (this wear mode is commonly observed to be dominant in the drilling of CFRPs). Fresh tool and severe wear (flank = 0.3 mm [25]) were tested in order to study different conditions for cutting forces and torque, and, in consequence, different levels of generated heat. Obtaining controlled worn geometries directly from wear tests is not easy, thus, the flank at the clearance surface was artificially generated by grinding.

**Table 1:** Mechanical properties of AGP 193-PW/8552 composite material [24].

Property	Value
Density, ρ (kg/m^3^)	1570
Resin content (%)	55.29
Longitudinal modulus, *E*_1_ (GPa)	68
Transverse modulus, *E*_2_ (GPa)	68
Major Poisson’s ratio, ν_21_	0.21
Longitudinal tensile strength, *X*_T_ (MPa)	880
Longitudinal compressive strength, *X*_C_ (MPa)	880
Transverse tensile strength, *Y*_T_ (MPa)	880
Transverse compressive strength, *Y*_C_ (MPa)	880
In-plane shear strength, *S*_T_ (MPa)	84

Several drilling experiments were performed in the ranges of cutting speed (25, 50, 100 m/min) and feed (0.05, 0.1, 0.15 mm/rev). From the observation of force and torque evolution, the tests corresponding to cutting speed 50 m/min and feed 0.1 mm/rev, with fresh and worn tool, were selected. In the selected cases, it was possible to identify steady values of force and torque in the different states of the drilling process and levels of energy large enough to consider the possibility of thermal damages in the matrix in the case of worn tool.

The characteristics of the workpiece and drill, stated for the experiments, allowed the calculation of time intervals defined in Equations (5)–(7). Accounting for the drill tip angle equal to 118°, the thickness of the composite plate and the drill radius equal to 6 mm, *t*_1_ = 0.38 s; *t*_2_ = 0.88 s; and *t*_3_ = 1.26 s.

**Figure 4:**
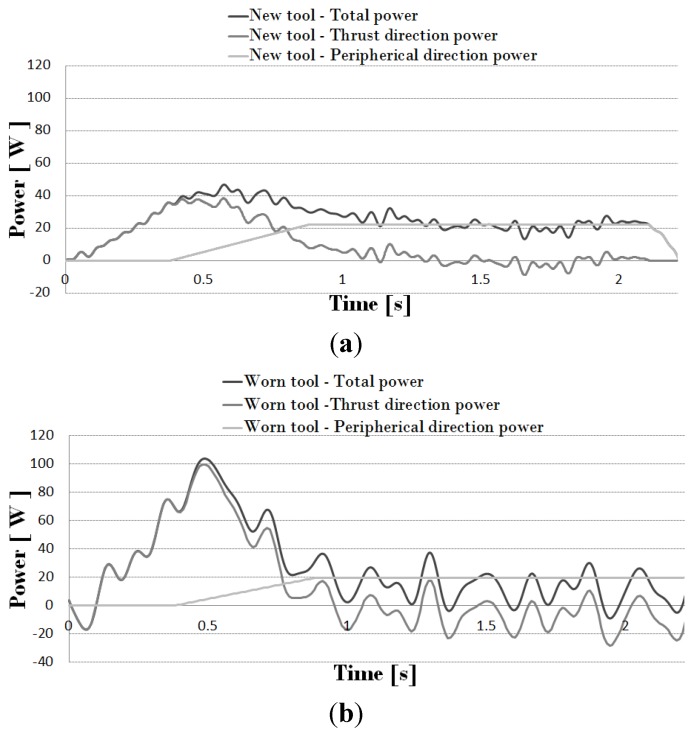
(**a**) Power due to the drilling operation (spindle velocity 2653 rpm and feed 0.1 mm/rev) for a new tool; (**b**) power due to the drilling operation (spindle velocity 2653 rpm and feed 0.1 mm/rev) for a worn tool (with flank = 0.3 mm).

As feed and rotary velocities are known, the evolution of thrust force and torque allowed the calculation of consumed power in penetration and rotation movement. In Figure 4, the total consumed power in both thrust and cutting movement (for cutting speed 50 m/min and feed 0.1 mm/rev) is presented. The curves were obtained with a fresh tool (Figure 4a) and a worn tool (Figure 4b), respectively.

From the recorded signal, the amount of heat generated due to friction was obtained. The power consumed by peripheral friction was calculated as the average value of total power once the exit stage is reached (*t* > *t*_3_). During entrance stage (*t* < *t*_1_) peripheral friction power (due to contact between the drill body and the hole wall) is null while during steady stage (*t*_1_ < *t* < *t*_2_) it varies linearly from 0 to the exit stage value. The power consumed by cutting edge friction was estimated according to Equation (3) as the total power, minus the energy consumed by composite breakage (Equations (5)–(7)), and minus the peripheral friction. Negative values in some points are the result of noise and also due to subtracting the peripheral power from the total power (as it was considered as a constant value when all the drill nose get through the specimen thickness, when this value is subtracted from the total power negative values appear but these values are not applied to the model since there is no material in the nose direction).

The discretization of the curve of power *vs.* cutting time, allows the statement of thermal flux to the workpiece and the analysis of heat propagation in the finite element code.

The heat propagation in the workpiece is analyzed in the next section.

## 3. Numerical Modeling of Temperature Distribution

The numerical model was developed using the commercial finite element code ABAQUS/Explicit [26]. The aim of the model is the analysis of heat propagation during the drilling process in order to identify critical zones with excessive temperature level.

Simplifying hypotheses have been formulated. First of all, the model does not account for chip removal; in fact, it is an uncoupled thermal model. The assumption of axial-symmetry was considered in order to create a simplified model with reasonably computational cost. It is worth noting that CFRP strength and thermal conductivity is orientation dependent, but the disposal of low computational cost models to evaluate heat generation from experimental data requires the assumption of a strong hypotheses. Unidirectional tape laminates present high anisotropy in mechanical and thermal properties, thus, a woven laminate was selected to minimize the effect of the anisotropy in the axial-symmetrical model.

Complex models of composite drilling recently developed in the literature (see, for instance, [7]) involve elevated computational costs. These models account for chip removal and are able to predict mechanical damage at the machined surface, both intra laminar and delamination. However, up to the present, mechanical analysis has not been coupled with heat generation and propagation. The main objective of the model developed in this paper is the prediction of thermal issues during drilling, however, mechanical damage cannot be predicted.

The model has been divided in zones corresponding to each drill revolution corresponding to penetration equal to the feed (this depth of penetration per revolution will be used as time increment per step to apply the loads).

The frictional heat generated in each time increment was calculated from the analysis explained in the previous section. The proportion of the frictional heat energy allocated to the chip is characterized by the coefficient of heat partition. In the case of composite cutting the chip is highly fragmented as it is not observed the adhesion mechanism at the tool/chip interface found in metal cutting, characterizing the sticking/sliding zones (see for instance [22]). The amount of heat transferred to the chip is neglected and the frictional heat is assumed to be transferred 50%/50% to the workpiece and to the tool. The present paper is one of the earliest works dealing with the thermal effects in composite cutting. Further improvements of the research in this field are desirable and the statement of heat partition in composite cutting should be soundly analyzed. It is worth noting that the nature of both materials in contact (composite and tool material) could cause the increase of the amount of heat transferred to the tool and, thus, the extension of the thermal affected zone of the workpiece would diminish. Simulations with lower level of percentage of heat transferred to the workpiece were carried out (25% and 40%, respectively). In the first case critical temperature were not reached, even in the case of worn tool. For the second case, the thermal damage appeared but the affected zone was smaller than in the case corresponding to 50%. Heat partition, 50%/50%, could be treated as a starting point for the analysis of thermal problems in composite cutting and an upper limit for the generation of damage.

**Figure 5:**
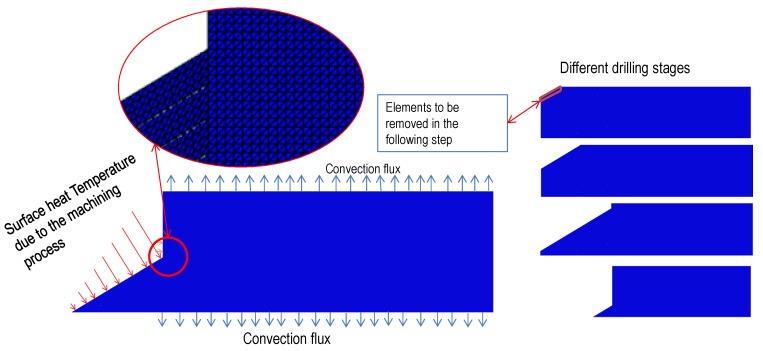
Scheme of the numerical model.

The scheme of the model is shown in Figure 5, including boundary conditions and geometry. The model was meshed with 70,000 linear triangular elements with a size of 25 μm. With the element size used in the model, each simulation takes around 2 h, being a reasonable computational cost. The element size was stated after several iterations; no change in the temperature distribution was observed when the element size was lower than 25 μm, however the computational cost increased.

The mechanical properties of the workpiece are summarized in Table 1. Thermal properties for CFRPs are given in a wide range in the literature, thus, the values used in this work (thermal conductivity 5 W/mK and specific heat 1100 J/KgK) were averaged from several references covering different applications [4,8,21,27,28].

The procedure of the simulation is described in the following. In a generic time step (being the time involved in a drill revolution) the amount of heat along the cutting edge and lateral wall is calculated, applying the analytical model to the measured thrust force and torque, as it was explained in the previous section. At the end of each step, the layer of elements corresponding to the chip area removed in one revolution of the drill is eliminated from the model (thus, the layer become inactive for heat propagation) and the heat corresponding to the subsequent step is applied.

## 4. Results and Discussion

The numerical model was applied to the analysis of heat propagation. The model developed was used for analysis of the effect of tool wear. The effect of wear on torque and thrust force were included in the estimation of heat generated, and the numerical model allowed the estimation of the temperature distribution and the establishment of critical levels.

**Figure 6:**
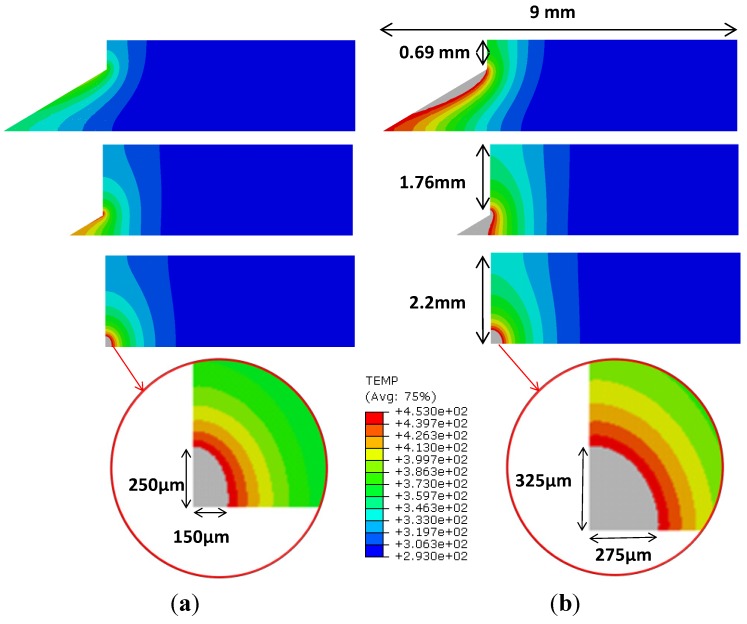
Predicted temperature (K) for tests developed at cutting speed 50 m/min and feed 0.1 mm/rev (grey zone represents temperature higher than 180 °C, 453 K): (**a**) fresh tool; (**b**) worn tool.

Figure 6 shows the evolution of the temperature fields as the entrance of the drill progresses. The cases, shown in Figure 6, correspond to drilling tests performed at cutting speed 50 m/min and feed rate 0.1 mm/rev. Figure 6a,b present, respectively, a fresh drill and a worn tool. It is observed at three stages of the drill entrance, the maximum level of the wall temperature occurred at the exit of the hole. It is worth noting that this zone also experience mechanical delamination [7]. Both effects would superpose inducing combined thermal and mechanical damage.

For a typical epoxy-based CFRP material, the initiation of resin degradation can be produced at a temperature of approximately 180 °C. The thermal damage at this temperature can create cracks leading to the onset of delamination and strength reduction [8,29]. The maximum temperature at the wall was significantly higher in the case of worn tool, with a temperature level higher than 180 °C (453 K) in a more extended area (in depth penetration below the machined wall equal to 275 μm). It is clear that this value of wear produces unacceptable level of temperature. In the case of a new tool the area reaching this high value of temperature is also significant (penetration beneath machined surface 150 μm). Although it is not directly comparable, the temperature distribution predicted with the model is in the order of that measured in milling CFRP [8]. The temperature at 0.3 mm beneath the machined surface reached 104 °C being drilling more critically under the thermal point of view.

## 5. Conclusions

This work focuses on the prediction of temperature at the workpiece during drilling of woven CFRPs composites. The approach combine experimental testing (to establish the evolution of thrust and torque with cutting time); analytical modeling to estimate the heat flux at the interface tool/workpiece, and numerical simulation to analyze the heat propagation and the maximum level of temperature in the workpiece. The main contribution of the work is the development of the combined approach for the prediction of thermal damage.

The model was applied to two different real cases of drilling: fresh tool and worn tool (significant level of flank wear). The numerical model showed the maximum temperature occurring at the hole wall, close to the exit of the drill, being a zone where mechanical delamination is commonly observed. The occurrence of thermal damage, in the case of excessive wear, enhances the risk of defect at the exit of the hole.

The model is simple and very efficient from the computational point of view. The problem of using realistic models of drilling, including penetration and cutting movement, and elements erosion is the computational cost. The implementation of simulations tools in industry for assistance during manufacturing requires rapid response. The model proposed could be easily implemented to detect excessive levels of thermal power, because of inappropriate cutting parameters or excessive wear of the tool.
